# Temperature-Dependent Tethered Locomotion Behavior in the Madagascar Hissing Cockroach Using a Controlled-Environment Treadmill Platform with Exploratory Illumination Assays

**DOI:** 10.3390/biology15120947

**Published:** 2026-06-17

**Authors:** Eduardo Gracidas-Reyes, Gerardo Diaz-Arango, Hector Vazquez-Leal, Francisco Marroquin-Gutierrez

**Affiliations:** 1 Departamento de Computación, Electrónica y Mecatrónica, Universidad de las Américas Puebla, Ex hacienda Sta. Catarina Mártir S/N, San Andrés Cholula 72810, Puebla, Mexico; 2 Facultad de Instrumentacion Electronica, Universidad Veracruzana, Cto. Gonzalo Aguirre Beltran S/N, Xalapa 91000, Veracruz, Mexico; guda.diaz.gd@gmail.com (G.D.-A.); hvazquez@uv.mx (H.V.-L.); 3 Coordinación de Maestría en Ingeniería Aeroespacial, Universidad Politécnica Metropolitana de Hidalgo, Ex Hacienda San Javier, Tolcayuca 1009, Tolcayuca 43860, Hidalgo, Mexico; fmarroquin@upmh.edu.mx

**Keywords:** *Gromphadorhina portentosa*, neuroethology, insect locomotion, thermal behavior, light behavior, environmental control, spherical treadmill

## Abstract

Quantitative analysis of insect locomotion is important for understanding how environmental variables shape behavioral output and for developing reproducible experimental paradigms in neuroethology and biohybrid research. In this study, locomotor responses of the Madagascar hissing cockroach (*Gromphadorhina portentosa*) were evaluated across a thermal range from 18 to 38 °C using an integrated low-cost platform combining a spherical treadmill motion-tracking system with synchronized environmental regulation of temperature and illumination. The results suggest a possible non-linear relationship between chamber temperature and locomotor activity. Thermal conditions below the programmed 30 °C chamber temperature were generally associated with higher cumulative activity and elevated peak activity-rate values but not low inactivity proportions. In contrast, higher temperatures were associated with more fragmented locomotor patterns characterized by preserved peak activity but increased inactivity proportions. Complementary repeated-measures illumination assays suggested preliminary UV-associated shifts in locomotor output under the tested treadmill conditions. Together, these findings provide a quantitative framework for studying environmentally modulated locomotion in *G. portentosa*.

## Simple Summary

Insects are highly sensitive to environmental conditions such as temperature and light, which can influence how they move and interact with their surroundings. Understanding these effects is important for studying animal behavior and for developing new experimental tools. In this study, we used a controlled laboratory platform that combines environmental regulation with a treadmill-based motion-tracking system to measure the tethered locomotor activity of Madagascar hissing cockroaches. We evaluated how movement changed across different temperature values and explored the effects of different lighting conditions. The results showed that tethered locomotor activity varied with temperature and that conditions around 30 °C were associated with a possible favorable overall locomotor profile under the tested experimental conditions. Higher temperatures were linked to more fragmented patterns of activity, with longer inactive periods. Exploratory light experiments also suggested that ultraviolet illumination may influence locomotor behavior. These findings provide new information about how environmental factors affect Madagascar hissing cockroach movement and demonstrate the usefulness of controlled environmental platforms for studying animal behavior. Such approaches may support future research in behavioral biology, environmental physiology, and bioinspired technologies.

## 1. Introduction

Insect locomotion is characterized by high efficiency and adaptability, emerging from the integration of diverse sensory inputs with coordinated motor patterns generated by compact neural circuits [1]. Because locomotor output reflects the interaction between neural control, biomechanics, and environmental context, insects have long served as powerful models for investigating sensorimotor integration and behavior under controlled experimental conditions. In this context, reliable quantification of locomotion is essential for understanding how environmental variables shape neurobehavioral output.

Among the many abiotic factors that influence insect locomotion, temperature and light are particularly relevant because both can strongly modulate the physiology and behavior of terrestrial ectotherms. Thermal variation affects metabolic rate, neuromuscular performance, sensory responsiveness, and overall locomotor capacity, thereby shaping activity patterns, ecological niche use, and behavioral performance [2,3,4]. Because many insects inhabit micro-climates where temperature fluctuates at fine spatial scales, understanding thermal sensitivity is essential for interpreting behavioral ecology and predicting performance under natural or changing environmental conditions [5]. These considerations are increasingly relevant as climate variability alters the frequency and magnitude of micro-habitat thermal shifts. Moreover, as ectotherms, insects exhibit strong temperature dependence in motor performance, sensory processing, and behavioral output [6], with locomotor activity often increasing with body temperature within an optimal range and then declining under more stressful thermal conditions [7,8].

Light also plays an important role in insect behavior, although its effects are often species-specific and depend on wavelength, intensity, and exposure conditions. Light cues can influence orientation, shelter seeking, foraging, escape responses, reproduction, and locomotor activity. For example, in the plant grasshopper *Nilaparvata lugens*, green light has been found to negatively affect both locomotion and growth [9]. Similarly, light has been reported to significantly affect the reproductive performance and locomotor behavior of the predatory flower bug *Orius sauteri* [10]. These findings suggest that illumination can act not only as a sensory stimulus but also as an environmental modulator of insect activity.

Cockroaches are particularly suitable for studying locomotor responses to temperature and illumination because of their robustness, behavioral plasticity, and reliance on diverse micro-habitats. Found across tropical and subtropical ecosystems, their behavioral responses, including shelter seeking, foraging, and activity modulation, are strongly influenced by local thermal and humidity conditions [11,12]. Despite this, quantitative studies focusing on temperature-dependent locomotion remain limited for several cockroach species, leaving an incomplete picture of how different cockroaches cope with thermal heterogeneity and how environmental variables interact to shape locomotor output.

One such species is *Gromphadorhina portentosa* (*Blattodea: Blaberidae*), the Madagascar hissing cockroach, which inhabits the understory of humid tropical environments and is commonly associated with leaf-litter micro-habitats [13,14]. *G. portentosa* is a large, wingless cockroach with a heavily sclerotized body and distinctive hissing acoustic displays used in defensive, social, and reproductive contexts [15,16]. Its size, robustness, ease of maintenance, and experimental tractability have contributed to its increasing use in physiological, behavioral, and bioengineering studies [17,18].

Previous studies have employed *G. portentosa* in a variety of experimental paradigms, including wireless stimulation, onboard sensing, remote modulation of locomotion, and cyborg or biohybrid applications [18,19,20,21]. These studies have shown that locomotor behavior in this species can be influenced through sensory or neural inputs, but they have also highlighted persistent challenges related to locomotor variability, inconsistent responsiveness, and difficulties in standardized behavioral quantification [22,23]. These limitations are especially relevant when the goal is to determine how environmental variables, rather than direct stimulation alone, modulate locomotor performance.

The role of illumination in cockroach locomotion further illustrates this complexity. Different cockroach species show distinct phototactic and wavelength-dependent responses. In *Periplaneta americana*, ultraviolet (UV) light has been observed to inhibit locomotion [24], and occlusion of the ocelli results in reduced locomotion [25]. In *Blatta lateralis*, blue light acts as a repellent, while red light serves as an attractant [26]. In other cockroach species, light-driven responses also depend on stimulus properties; for example, *Periplaneta americana* shows stronger optomotor reactions under brighter light and with larger visual stimuli, indicating reliance on spatial summation to maintain vision in dim conditions [27]. Although a comprehensive understanding of *G. portentosa* light behavior is still lacking, previous work has used light stimulation, including UV light, to modulate locomotion [28], offering a non-invasive alternative to antennal methods. However, the reliability of light-induced locomotor modulation in *G. portentosa* remains uncertain, particularly because some cockroach species, such as *Eublaberus posticus*, can become habituated to repeated light exposure [29].

Temperature-dependent locomotion represents an equally important but still underexplored aspect of *G. portentosa* behavior. Studies in other insects, including *Acheta domesticus*, *Drosophila*, and *Sitona gressorius*, have shown that temperature strongly affects activity, speed, and locomotor performance [30,31,32]. However, comparable quantitative data for *G. portentosa* remain scarce, limiting our ability to understand how temperature shapes its behavioral ecology and constraining applied research that relies on predictable locomotor dynamics. Establishing these baselines is therefore relevant not only for comparative physiology and behavioral ecology but also for technological applications in which insect locomotion is used as inspiration or as a biological substrate for control systems and robotics [18,19,33].

A major obstacle to characterizing these environmental effects is methodological. Common approaches for quantifying insect locomotion include circular arenas monitored by video tracking, inertial measurement units, and hybrid sensing systems [33,34]. Although effective in many contexts, such setups often require large experimental spaces, computationally intensive video processing, or external sensor payloads that may alter natural locomotion. Depth-sensing approaches, including Kinect-based systems, alleviate some of these limitations but generally lack the environmental regulation and mechanical stability required for standardized neuroethological or stimulation-based experiments [35]. In this context, spherical treadmill systems provide an alternative strategy by allowing animals to walk while remaining spatially stationary, enabling continuous measurement of locomotor output with minimal physical constraint. In *G. portentosa* and other insects, treadmill implementations based on optical or mechanical sensing have demonstrated the utility of these systems for locomotor measurements while also highlighting trade-offs in cost, complexity, and environmental control [22,36,37].

We investigate how environmental temperature and illumination modulate the locomotor activity of *G. portentosa*. To quantify these effects under controlled conditions, we used an integrated low-cost spherical treadmill platform with synchronized regulation and monitoring of temperature and illumination. Using this framework, we evaluated locomotor behavior across a thermal gradient from 18 to 38 °C and performed complementary illumination assays to explore light-associated locomotor modulation under controlled experimental conditions. The main objective of this study is to provide quantitative behavioral data on the environmental sensitivity of *G. portentosa* tethered locomotion, with particular emphasis on thermal modulation and exploratory illumination responses.

This study is organized as follows: Section 2 describes the methodological platform used to acquire the experimental data, the animal preparation procedure, the extracted locomotor parameters, and the statistical analysis methods. Section 3 presents the results of the exploratory illumination assays, the temperature-dependent locomotion patterns, and the statistical analyses. Section 4 discusses these findings and their limitations and compares them with the current literature. Finally, Section 5 summarizes the main conclusions of the study and discusses proposed directions for future work.

## 2. Materials and Methods

### 2.1. Environmental Control Chamber

The environmental control chamber consisted of a black plastic enclosure internally lined with insulating protective foam and sized to house both the spherical treadmill and the experimental subject. The chamber had internal dimensions of approximately 54.1 × 45.0 × 30.0 cm (L × W × H). Chamber temperature was regulated bidirectionally using two 12 V, 3 A thermoelectric Peltier modules, driven by an ESP32-based proportional-integral-derivative (PID) controller through a dual-channel high-power motor driver, see Supplementary Materials for more information about the thermal characterization. To improve environmental monitoring within the chamber, temperature and humidity were measured using redundant sensors, including SHT45 units and a BME680 module, whereas illumination was monitored using GY-30 light sensors. These components were bought from MOUSER ELECTRONICS, Moctezuma, Mexico. The chamber illumination system comprised four RGB LEDs and four ultraviolet LEDs positioned at the corners of the lateral walls to promote a uniform light distribution across the experimental space. The overall specifications for each light source (according to the manufacturer STEREN, Mexico City, Mexico) are summarized in Table 1. However, irradiance and spectral power distribution were not independently measured so specific experimental wavelength values were unknown. Light sources were independently switched through a four-channel relay module, and the entire environmental control system was powered by a single 12 V, 10 A supply. Under standard room conditions (22–26 °C), the chamber was capable of reaching temperatures from approximately 18 to 40 °C with an average stationary error rate of approximately ±0.25 °C.

### 2.2. Motion-Tracking System

**Figure 1:**
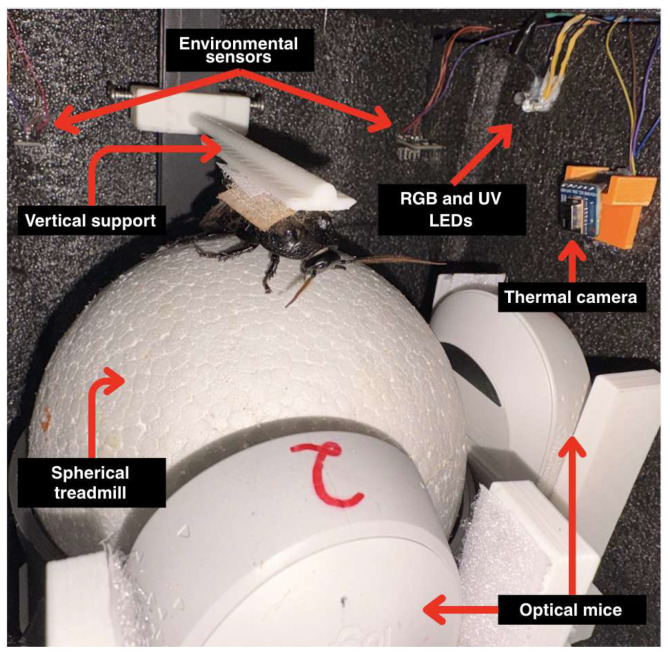
Photograph of the integrated motion-tracking setup inside the environmental chamber. The system includes the spherical treadmill, the adjustable vertical support used to position the animal, the optical motion sensors, the environmental sensors, the illumination modules, and the thermal camera.

Locomotor activity was recorded using a spherical treadmill built around a custom 3D-printed PLA support structure and a 12 cm diameter ball mounted on low-friction bearings. Each individual was tethered to the vertical support using a Velcro attachment fixed to the thorax, allowing the cockroach to perform walking-related locomotion while remaining spatially stationary. Ball rotation was measured using two Logitech M170 optical mice (1000 DPI) mounted on adjustable 3D-printed holders and positioned orthogonally at 90° with respect to each other, following the general sensing logic of previous treadmill-based systems for insect locomotion [22]. No external motion-capture calibration was performed; therefore, the resulting displacement variables should be interpreted as treadmill-derived locomotor output rather than absolute free-walking displacement. The integrated arrangement of the treadmill, optical sensors, environmental modules, and vertical support is shown in Figure 1.

The orthogonal arrangement enabled independent detection of motion components along two planes, here referred to as lateral and frontal axes, from which a combined locomotor signal was reconstructed. To ensure proper alignment and minimize mechanical interference, both the mouse holders and the vertical animal-support platform were designed to be adjustable.

### 2.3. Data Acquisition and Synchronization

**Figure 2:**
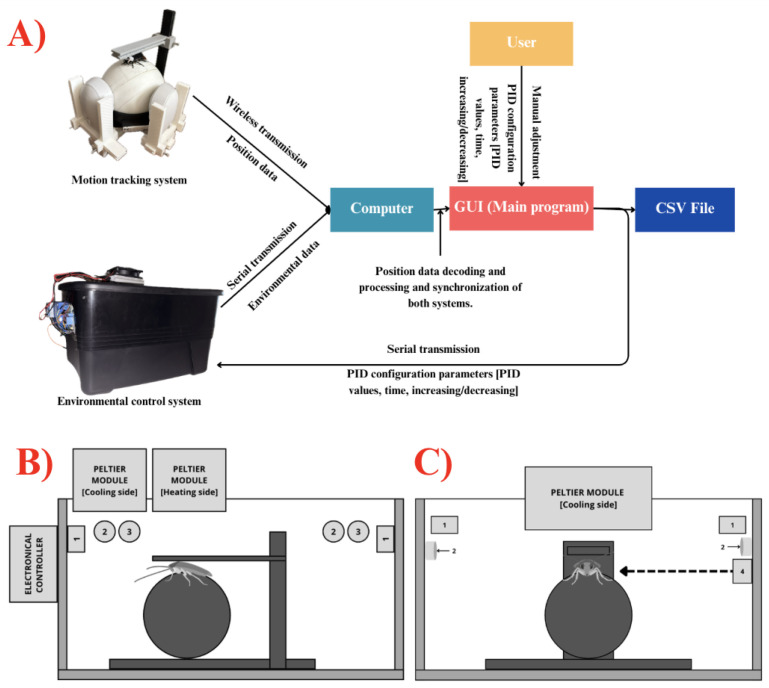
(**A**) Communication diagram illustrating data exchange between the motion-tracking subsystem, the environmental control module, and the graphical user interface (GUI) on the software side. (**B**) Lateral schematic view of the integrated environmental control and motion-tracking system. (**C**) Frontal schematic view of the same system. In panels (**B**,**C**), labels indicate component placement: 1, environmental sensors (SHT45, BME680 or GY-30); 2, RGB light sources; 3, ultraviolet (UV) light sources; and 4, infrared thermal camera.

The environmental control and motion-tracking subsystems were synchronized using a custom software environment running on an external computer, as shown in Figure 2 (version 0.2: https://doi.org/10.5281/zenodo.17941650). The ESP32 firmware continuously acquired temperature, humidity, illumination, and thermal-camera data, while also controlling the actuators for temperature regulation and illumination. Environmental measurements were sent to the computer through serial communication. At the same time, locomotor data were collected from two wireless optical mice through a USB-connected receiver, allowing simultaneous acquisition of incremental displacement signals from two orthogonal sensing planes.

**Figure 3:**
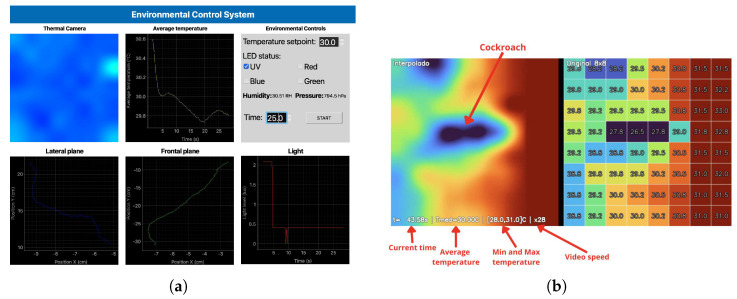
Software outputs generated during experimental trials. (**a**) The graphical interface displays the thermal-camera feed together with real-time plots of locomotor activity, temperature, and illumination, and provides controls for setting the target temperature and illumination conditions. (**b**) The thermal-video output (at t=43.58 s) shows an interpolated image in which the cockroach shape is visible together with the heatmap and temperature values of the current frame captured by the thermal camera. Red colors indicate temperature values at or substantially above 30 °C, whereas blue colors indicate temperature values substantially below 30 °C.

A custom Python-based acquisition interface synchronized and stored the environmental and locomotor data streams in real time. During each trial, the software displayed live plots of motion, temperature, and illumination together with the thermal-camera feed, while also providing controls for defining the target temperature and enabling or disabling individual light sources when required as illustrated in Figure 3a.

The software additionally generated a thermal video during each experimental procedure in order to obtain approximate surface-temperature estimates of the cockroach throughout the trial as shown in Figure 3b. These measurements were used only as a qualitative reference to verify that the animal remained within the expected thermal range of the chamber conditions. Because the thermal camera has a manufacturer-reported precision of approximately ±2.5 °C, the resulting thermal images were not interpreted as precise measurements of internal physiological body temperature.

Once initiated, the system automatically maintained the programmed environmental conditions and continuously stored all incoming measurements in CSV format for subsequent analysis. Experimental sessions lasted approximately 25 min, ensuring that locomotor activity and environmental variables were recorded within the same synchronized dataset.

### 2.4. Animal Preparation

Experimental subjects were selected from a laboratory colony of *Gromphadorhina portentosa* maintained under stable environmental conditions with ad libitum access to food (carrots, zucchini and dog food) and water. Unlike pest cockroach species of medical concern, *G. portentosa* presents comparatively low sanitary risk to humans, which further supports its usefulness as an experimental organism [38,39]. In addition, experimental guidelines for invertebrates emphasize minimizing discomfort during handling and manipulation [40].

To reduce excessive morphological variability during treadmill experiments, only individuals with a body length of at least 5 cm were included. Body length was measured from the anterior margin of the pronotum to the posterior end of the abdomen using digital calipers. Sex was determined based on abdominal morphology following standard descriptions for the species. Exact chronological age was not known; therefore, body length and sex were recorded as available biological descriptors, and age-related effects could not be explicitly modeled. The final thermal dataset included 28 females and 14 males. Body length ranged from 5.0 to 7.1 cm, with a mean ± SD of 5.98 ± 0.486 cm.

**Figure 4:**
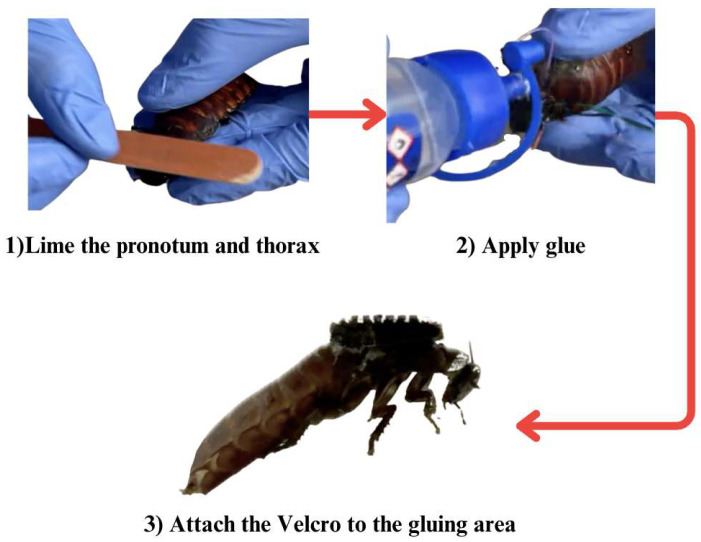
Preparation procedure for tethering *G. portentosa* to the vertical support. The pronotum and thoracic dorsal surface were gently abraded, adhesive was applied to the prepared area, and a Velcro fragment with individual identification was attached before the recovery period.

Prior to behavioral testing, each animal was prepared for tethered locomotion by gently abrading the dorsal surface of the pronotum and adjacent thoracic region with fine sandpaper, following handling considerations commonly used for external attachment in this species [41]. The complete preparation procedure is summarized in Figure 4. Once the attachment area was prepared, a small amount of cold silicone adhesive was applied together with a Velcro fragment containing an individual identification mark. This procedure enabled each cockroach to be coupled to the adjustable vertical support during experimental sessions while maintaining stable positioning over the spherical treadmill.

All handling and preparation steps were carried out carefully to minimize stress, discomfort, and post-manipulation behavioral alterations, in accordance with general welfare recommendations for invertebrate animals [40]. Nevertheless, because tethering and handling may influence behavioral state and locomotor performance, the resulting measurements should be interpreted as treadmill-based locomotor output under controlled experimental conditions.

After the attachment procedure, individuals were housed in small groups within plastic containers and allowed to recover for at least 12 h before testing in order to reduce potential short-term effects of handling, manipulation, and social disturbance on subsequent locomotor performance [16].

As no untethered open-arena control was included in the present study, the results should not be interpreted as direct measurements of free-walking behavior, but rather as tethered treadmill-based locomotor output under controlled environmental conditions. Additionally, no mortality occurred during the experimental procedures, and no individuals were excluded because of failed tethering preparation.

### 2.5. Experimental Design

The experimental design comprised two complementary protocols. The main protocol evaluated temperature-dependent locomotion using 42 randomly selected *G. portentosa* individuals tested under six programmed chamber-temperature conditions ranging from 18 to 38 °C. Thus, the thermal experiment followed a repeated-measures design in which each individual experienced all temperature conditions. Each experimental session lasted approximately 25 min, allowing an initial acclimation period together with a sufficiently long recording window to capture representative treadmill-based locomotor activity. During each trial, locomotor displacement and environmental variables were recorded continuously.

To reduce potential fatigue, habituation, and order effects, temperature-session order was assigned using a constraint-based pseudo-random scheduling algorithm. At each scheduling step, only individuals that had completed the minimum 2 h resting interval were eligible for a new trial. During the resting intervals, individuals were returned to their colony, where they had access to shelter, food, and water. For each eligible individual, the next temperature condition was selected pseudo-randomly among the remaining temperature conditions for that individual, avoiding a fixed temperature sequence. This procedure reduced the likelihood that a specific temperature was systematically associated with trial order or time of day. Illumination during the temperature experiments was maintained under standardized low-light conditions (<30 lux) to minimize possible light-associated modulation of locomotor behavior.

Because each of the 42 individuals was tested once under each of the six programmed chamber-temperature conditions, the thermal protocol comprised 252 planned trials. Experimental sessions were distributed across multiple testing days. Only the experimental assay for cockroach 22 at 30 °C was removed from further analysis since it was not saved properly by the system due to an error in the pipeline of the platform, leaving 251 trials for analysis.

**Figure 5:**
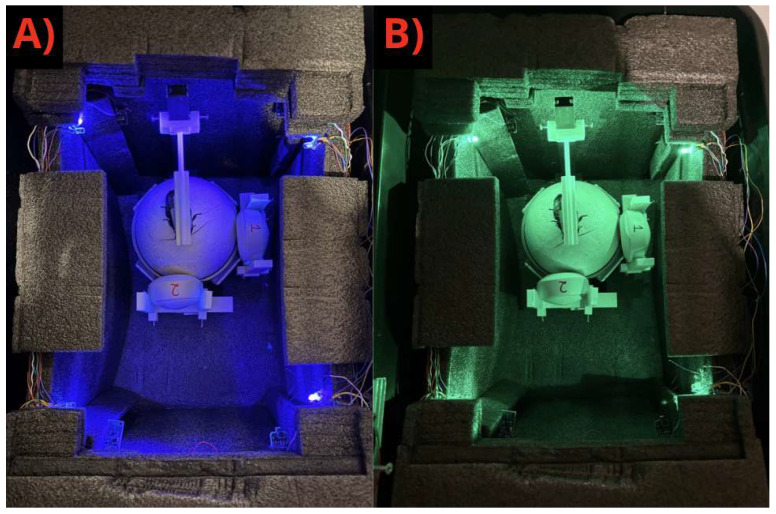
Representative illumination conditions generated inside the environmental chamber during the complementary light-modulation assays. (**A**) Ultraviolet illumination. (**B**) Green illumination. The no-light condition was used as the dark control during the baseline phase and in the control group.

A second protocol was incorporated as an exploratory proof-of-concept assay to evaluate the sensitivity of the platform to controlled illumination changes. For this complementary experiment, 12 additional cockroaches were tested under all three light conditions: ultraviolet (UV), green light, and no-light control (NL), as shown in Figure 5. In all cases, trials began with a 10 min baseline period under no-light conditions, followed by a second 10 min period corresponding to the assigned illumination condition. Thus, in the UV and green assays, individuals experienced a light transition after the baseline phase, whereas the no-light condition (NL) remained under complete darkness during both phases of the assay.

Because each illumination trial consisted of a baseline dark phase followed by an experimental phase, locomotor modulation could be quantified as an intra-individual pre/post change, allowing both absolute and relative changes in locomotor activity to be evaluated under each illumination condition.

### 2.6. Locomotion Signal Processing and Derived Metrics

**Figure 6:**
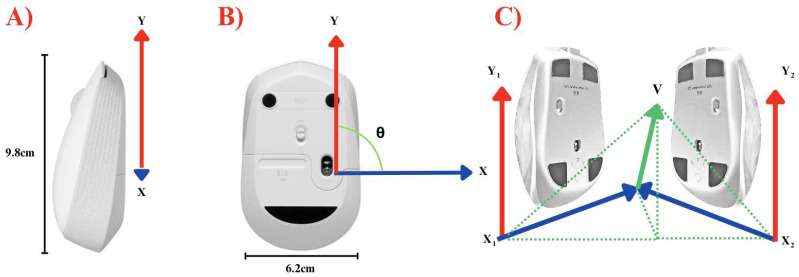
Schematic representation of locomotor signal reconstruction from the two orthogonal optical sensors. Each sensor records motion in an independent sensing plane, and both contributions are combined into a single resultant locomotor signal used for cumulative activity estimation. (**A**) Lateral movement plane of detecion. (**B**) Frontal movement plane of detection. (**C**) Representation of the combination of the frontal and lateral movement planes of detection to obtain the resultant vector indicative of locomotor activity.

Raw locomotor output was obtained from the two orthogonally oriented optical sensors as synchronized incremental displacements in two independent sensing planes. Because the animal remained tethered over a spherical treadmill rather than moving freely across a physical arena, the recorded signals were interpreted as a proxy of locomotor output rather than as an absolute reconstruction of translational displacement in Cartesian space. The contributions of both orthogonal optical sensors were combined into a single resultant locomotor signal, as schematically illustrated in Figure 6. For this reason, the primary descriptor extracted from the motion-tracking system was defined as *cumulative locomotor activity*, which integrates the total amount of ball rotation generated by the animal over time. At each sampling step, the incremental motion detected by the two optical sensors was combined according to:

(1) $$\Delta s_{i}=\sqrt{{\left(\Delta x_{1,i}\right)}^{2}+{\left(\Delta y_{1,i}\right)}^{2}+{\left(\Delta x_{2,i}\right)}^{2}+{\left(\Delta y_{2,i}\right)}^{2}},$$

where $\Delta x_{1,i}$ and $\Delta y_{1,i}$ correspond to the incremental motion recorded by the first optical sensor, and $\Delta x_{2,i}$ and $\Delta y_{2,i}$ to that recorded by the second sensor. The cumulative locomotor activity signal was then computed as

(2) $$A_{\mathrm{cum}}\left(t_{k}\right)=\sum_{i=1}^{k}\Delta s_{i}.$$

This variable was used as the main descriptor of overall locomotor engagement during the trial.No additional smoothing or digital filtering was applied before calculating derivative-based descriptors. The acquisition rate was approximately 28 Hz, which was sufficient to capture the treadmill ball displacements produced by cockroach locomotion under the present experimental conditions. Because derivative-based metrics can amplify sample-to-sample fluctuations, the activity modulation index was interpreted as an exploratory descriptor of temporal changes in locomotor output rather than as a direct physiological measurement of acceleration.

From the cumulative activity signal, two dynamic metrics were derived to characterize the temporal evolution of locomotor output. The first derivative of the activity signal was defined as the *instantaneous activity rate*, conceptually equivalent to velocity in classical kinematic terms, and was calculated using a central-difference approximation:

(3) $$R_{i}=\frac{A_{\mathrm{cum},i+1}-A_{\mathrm{cum},i-1}}{2\Delta t},$$

where Δt is the sampling interval. This variable quantifies the instantaneous rate at which locomotor activity accumulated and therefore captures moment-to-moment movement intensity. The second derivative was defined as the *activity modulation index*, conceptually analogous to acceleration:

(4) $$M_{i}=\frac{R_{i+1}-R_{i-1}}{2\Delta t}.$$

This descriptor reflects the temporal variation in activity rate and was used as an indicator of abrupt behavioral transitions, such as rapid movement initiation, sudden slowing, or short locomotor bursts. For both variables, absolute maximum values were extracted to estimate peak locomotor performance, and arithmetic means were computed across the full recording to summarize average locomotor intensity and average temporal modulation:

(5) $$\bar{R}=\frac{1}{N}\sum_{i=1}^{N}R_{i},\;\bar{M}=\frac{1}{N}\sum_{i=1}^{N}M_{i}.$$

To complement these primary measures, additional descriptors of locomotor organization were computed from the reconstructed activity sequence. First, the *inactivity proportion* was defined as the percentage of sampled time points in which no effective increase in cumulative locomotor activity was detected, providing an estimate of the fraction of the session spent immobile or behaviorally arrested. Second, a *path irregularity index* was calculated as the ratio between total accumulated activity and the straight-line displacement between the beginning and end of the reconstructed trajectory, thereby providing a dimensionless estimate of locomotor directness. Higher values of this index indicate more irregular or meandering movement patterns. The *turn index* was computed from changes in the direction of consecutive reconstructed displacement vectors and was used as an exploratory descriptor of reorientation-related locomotor output. Finally, the *exploration footprint* was estimated as the convex hull enclosing the reconstructed trajectory samples using the ConvexHull function from scipy.spatial, and was included as a compact descriptor of the spatial extent covered by locomotor output during the trial.

For the complementary illumination experiments, locomotor responses were additionally summarized using phase-comparison metrics derived from the two-segment structure of each trial, consisting of a 10 min dark baseline followed by a 10 min experimental phase. This design enabled each individual to serve as its own reference, allowing light-induced modulation to be quantified as a within-subject change. First, *net activity change* was calculated as the difference between cumulative locomotor activity measured during the second phase and that measured during the baseline phase:

(6) $$\Delta A=A_{\mathrm{post}}-A_{\mathrm{pre}}.$$

Positive values indicate an increase in total locomotor activity after the transition to the assigned condition, whereas negative values indicate a reduction. Second, *inactivity change* was defined as the difference in inactivity proportion between both phases:

(7) $$\Delta I=I_{\mathrm{post}}-I_{\mathrm{pre}}.$$

This metric was used to determine whether light exposure reduced behavioral arrest or, conversely, promoted immobility. In addition, a linear fit of cumulative locomotor activity as a function of time was computed separately for the pre-stimulus and post-stimulus phases, and the slope of each fit was interpreted as the *phase activity slope*, i.e., the average rate of activity accumulation during that interval. From these values, an *activity-rate shift* was calculated as

(8) $$\Delta S=S_{\mathrm{post}}-S_{\mathrm{pre}},$$

where $S_{\mathrm{pre}}$ and $S_{\mathrm{post}}$ are the slopes estimated for the baseline and experimental phases, respectively. Because absolute changes can depend strongly on the initial baseline level, relative modulation was also quantified using logarithmic post/pre ratios. For cumulative activity, the *relative activity gain* was defined as

(9) $$G_{A}=\log\left(\frac{A_{\mathrm{post}}+1}{A_{\mathrm{pre}}+1}\right),$$

where the constant 1 was added to avoid numerical instability in cases of very low activity values. Likewise, for the phase slopes, the *relative activity-rate gain* was computed as

(10) $$G_{S}=\log\left(\frac{S_{\mathrm{post}}+\varepsilon }{S_{\mathrm{pre}}+\varepsilon }\right),$$

where ε is a small positive constant introduced to avoid division by values close to zero. These logarithmic metrics were included to characterize multiplicative changes in locomotor output while reducing the asymmetry between increases and decreases. Together, the full set of descriptors allowed the behavioral response to be quantified not only in terms of overall activity magnitude but also in terms of temporal dynamics, immobility shifts, directional irregularity, exploratory spread, and intra-individual modulation induced by illumination changes.

### 2.7. Statistical Analysis

All statistical analyses were designed according to the structure of each experimental protocol and the type of locomotor descriptor extracted from the motion-tracking system. However, trial order was not included as an explicit statistical covariate, and residual order-related effects cannot be completely excluded.

For the thermal experiment, in which the same individuals were evaluated under the different programmed temperatures (from 18 °C to 38 °C), the effects of temperature on locomotor behavior were assessed using mixed-effects models. This approach was selected because it allows the effect of the fixed environmental factor to be estimated while accounting for repeated measurements and inter-individual variability among cockroaches. Programmed chamber temperature was treated as a fixed effect, while individual identity (ID) was included as a random intercept to account for repeated measurements obtained from the same cockroaches across thermal conditions.

Cumulative locomotor activity, instantaneous activity rate, and inactivity proportion were considered primary locomotor outcomes because they directly quantify overall locomotor performance. Additional descriptors including activity modulation index, path irregularity index, turn index and exploration footprint were treated as exploratory metrics intended to characterize higher-order locomotor dynamics.

Pairwise post hoc comparisons with Bonferroni correction for multiple testing were performed in order to identify the specific temperature groups that differed from one another. To further characterize the non-linear nature of the thermal response, Spearman rank correlation coefficients were also calculated between temperature and each locomotor variable over three thermal intervals: 18–30 °C, 30–38 °C, and the complete 18–38 °C range. Model assumptions were evaluated through visual inspection of residual-versus-fitted plots and Q–Q residual distributions to assess homoscedasticity and approximate normality. No additional variable transformations were required after residual diagnostics indicated acceptable model behavior.

In addition, an auxiliary mixed-effects analysis was performed to evaluate the contribution of sex and body length to locomotor variability. For this secondary analysis, sex was coded as a binary factor and body length was grouped using a 6 cm threshold, which was selected because it closely matched the mean body length of the thermal dataset (5.98 cm) and approximately separated individuals into smaller and larger body-size categories.

No statistical analysis was done for the complementary illumination assays due to the limited population. Because the illumination assay involved the same 12 individuals tested across all three light conditions, the analysis was based on within-subject contrasts and descriptive repeated-measures comparisons using boxplots to compare their means. Although this design reduces inter-individual variability relative to independent groups, the assay was still treated as exploratory because the total number of animals was limited and the protocol was intended as a proof-of-concept illumination assay rather than as a population-level characterization of spectral preference.

Throughout the analyses, statistical significance was evaluated using an α level of 0.05, and results were summarized using model-effect heatmaps, boxplots, and correlation summaries for the most informative locomotor descriptors.

All statistical analyses were performed in Python version 3.12 using the scipy, statsmodels, pandas, seaborn, and matplotlib libraries.

## 3. Results

### 3.1. Illumination Exploratory Results

**Figure 7:**
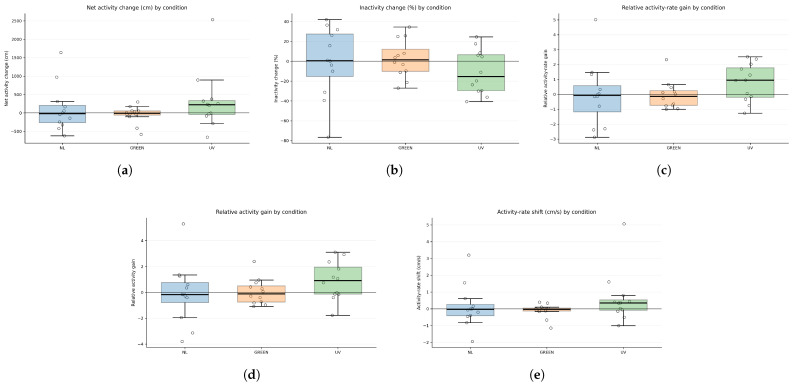
Exploratory illumination-response metrics comparing the 10 min baseline dark phase with the 10 min second experimental phase under no-light (NL), green-light, and ultraviolet (UV) conditions. (**a**) Net activity change by condition in cm. (**b**) Inactivity change by condition as percentage points. (**c**) Relative activity-rate gain by condition (dimensionless). (**d**) Relative activity gain by condition (dimensionless). (**e**) Activity-rate shift by condition in cm·s^−1^. In all boxplots, the central line indicates the median, boxes represent the interquartile range, whiskers indicate the non-outlier range, and individual points represent individual trials from the same 12 cockroaches tested under all three illumination conditions. Across metrics, the NL condition remained near zero, indicating little average change during the 20 min experimental session. Under green light, most metrics showed a slight decrease relative to baseline, whereas inactivity change increased slightly. The UV condition was associated with stronger responses, with increases relative to baseline in all metrics except inactivity change, whose mean was considerably below zero.

**Figure 8:**
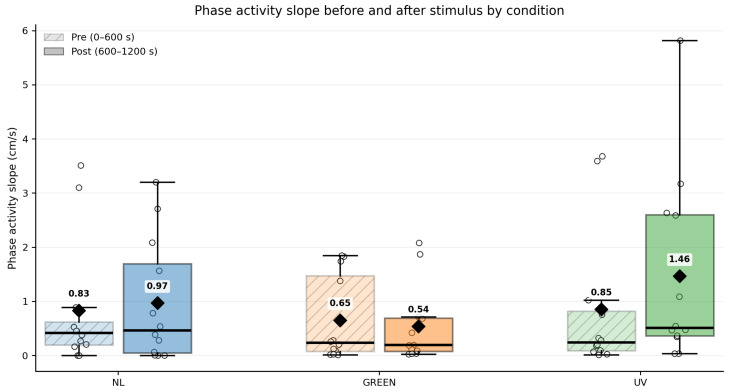
Phase activity slope before and after stimulus under no-light (NL), green, and ultraviolet (UV) conditions. The figure compares the average rate of cumulative activity increase during the baseline dark phase and during the second experimental phase of each trial. The central line indicates the median, boxes represent the interquartile range, whiskers indicate the non-outlier range, and individual points represent individual trials.

Exploratory within-subject comparisons between the baseline dark phase and the second experimental phase suggested a possible condition-dependent modulation of locomotor output. Ultraviolet illumination was associated with higher net activity change (Figure 7a), larger relative activity gain (Figure 7d), and higher relative activity-rate gain (Figure 7c) than the other conditions. It also showed the clearest tendency toward reduced inactivity after stimulus onset (Figure 7b) and a higher post-stimulus increase in activity slope (Figure 8) and activity-rate shift (Figure 7e). In contrast, the no-light condition remained centered near zero for most change-based metrics, whereas the green-light condition showed smaller shifts and a slight tendency toward reduced activity. The phase-based analysis further supported these differences. Under no-light conditions, mean activity remained nearly unchanged before and after the stimulus time point (t=600 s), although variability increased during the second phase (Figure 8). Green light produced a slight reduction in both mean activity and variability, suggesting a more inactive and homogeneous behavioral state. UV illumination, in contrast, showed a preliminary increase in locomotor output. However, this result should be interpreted cautiously because phase order was not counterbalanced.

### 3.2. Temperature-Dependent Locomotor Patterns

**Figure 9:**
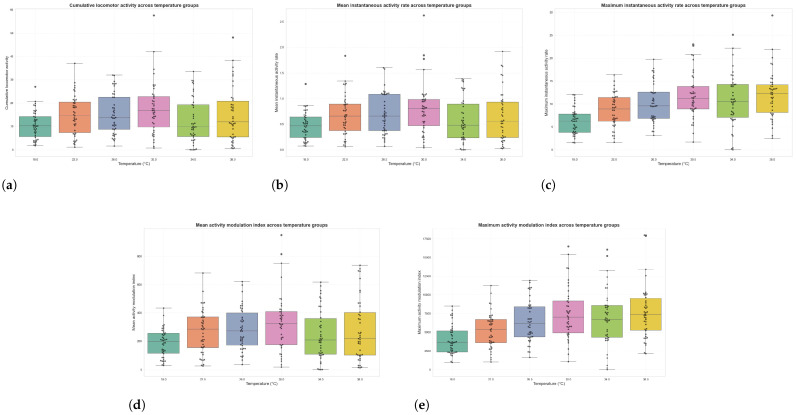
Temperature-dependent activity-intensity and modulation descriptors across the tested chamber-temperature groups. Panels show boxplots for (**a**) cumulative locomotor activity in cm, (**b**) mean instantaneous activity rate in cm·s^−1^, (**c**) maximum instantaneous activity rate cm·s^−1^, (**d**) mean activity modulation index in cm·s^−2^, and (**e**) maximum activity modulation index in cm·s^−2^. Each temperature condition includes repeated-measures observations from 42 individuals. In all boxplots, the central line indicates the median, boxes represent the interquartile range, whiskers indicate the non-outlier range, and individual black points represent individual trials. Overall, these metrics showed a broadly similar thermal pattern: values tended to increase with temperature up to approximately 30 °C, whereas above this point they either declined, with some metrics reaching their lowest mean values at 34 °C, or remained relatively unchanged.

The thermal experiments suggested a non-linear relationship between ambient temperature and treadmill-based locomotor performance in *Gromphadorhina portentosa*. Cumulative locomotor activity was associated with an increase from the lowest temperatures toward intermediate conditions, reaching its possible highest central tendency around 30 °C before declining or losing this favorable pattern at higher temperatures (Figure 9a). A similar trend was observed for maximum instantaneous activity rate, which increased from 18 to 30 °C (Figure 9c), and for mean instantaneous activity rate, although the latter showed a less pronounced and more variable pattern (Figure 9b). Together, these descriptors indicate that intermediate temperatures favored stronger locomotor bouts and, to a lesser extent, higher average locomotor intensity.

Modulation-related descriptors showed a comparable thermal profile. Maximum activity modulation index seemed to increase from colder conditions toward 30 °C (Figure 9e), while mean activity modulation index was associated with a broader and more variable increase across the lower-to-intermediate range (Figure 9d).

**Figure 10:**
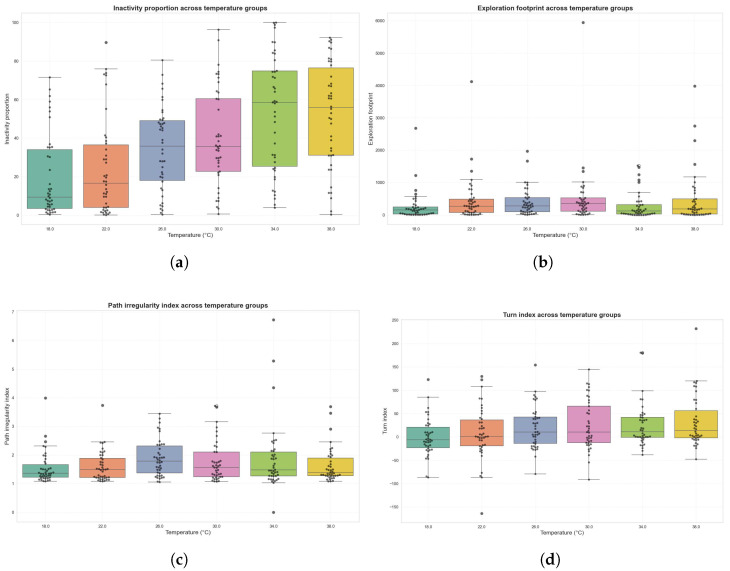
Temperature-dependent locomotor continuity, spatial-spread, and path-organization descriptors across the tested chamber-temperature groups. Panels show boxplots for (**a**) inactivity proportion in %, (**b**) exploration footprint in cm^2^, (**c**) path irregularity index (a.u), and (**d**) turn index (a.u). Each temperature condition includes repeated-measures observations from 42 individuals. In all boxplots, the central line indicates the median, boxes represent the interquartile range, whiskers indicate the non-outlier range, and individual black points represent individual trials.

The upper thermal conditions were mainly characterized by reduced locomotor continuity. Inactivity proportion reached its highest values at the warmer end of the gradient, particularly at 34 and 38 °C (Figure 10a). Path irregularity did not follow a monotonic pattern, although warmer conditions showed greater dispersion and reduced directional consistency in some cases (Figure 10c). Exploration footprint was generally broader near intermediate temperatures, especially around 30 °C, but showed substantial inter-individual variability (Figure 10b). Turn index also seemed to increase from lower temperatures toward the middle of the gradient, with 30 °C showing one of the widest and most elevated distributions (Figure 10d).

### 3.3. Mixed-Effects and Post Hoc Thermal Analysis

**Figure 11:**
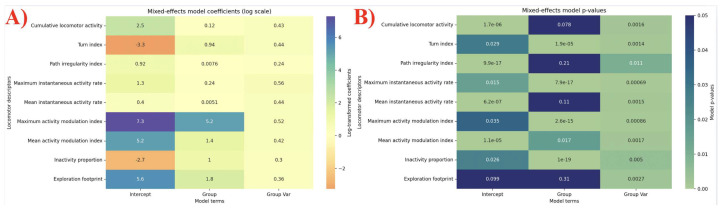
Mixed-effects analysis of temperature-dependent locomotor descriptors summarizing the magnitude, direction and statistical support of temperature related effects across the locomotor variables extracted from the motion-tracking system. (**A**) Log-scaled coefficients of the mixed-effects models fitted for each locomotor descriptor. (**B**) Corresponding model *p*-values for the same descriptors and model terms.

The mixed-effects analysis supported the results suggestions that temperature did not affect all locomotor descriptors with the same magnitude or consistency. Across the complete set of variables summarized in Table 2, the largest model coefficients seemed to concentrate in descriptors related to locomotor intensity and rapid temporal variation, particularly maximum activity modulation index, mean activity modulation index, maximum instantaneous activity rate, and exploration area (Figure 11A). However, statistical support was strongest for maximum activity modulation index, mean activity modulation index, maximum instantaneous activity rate, inactivity proportion, and turn index (Figure 11B). Cumulative locomotor activity remained close to the conventional significance threshold (p=0.078), whereas mean instantaneous activity rate, path irregularity index, and exploration footprint indicated weaker evidence across the full thermal range. This pattern suggest that temperature mainly affected peak output, temporal modulation, and behavioral arrest, rather than producing uniform changes across all descriptors.

**Figure 12:**
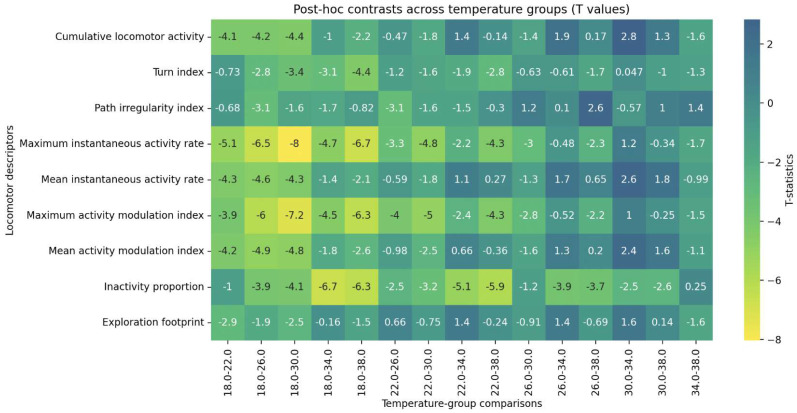
Post hoc contrasts across temperature groups for the locomotor descriptors extracted from the motion-tracking system. The heatmap shows T-values for pairwise comparisons between temperature groups, highlighting which descriptors contributed most strongly to the separation among thermal conditions. Stronger contrasts were concentrated in maximum instantaneous activity rate, maximum activity modulation index, and inactivity proportion, particularly around the transition toward intermediate temperatures.

**Figure 13:**
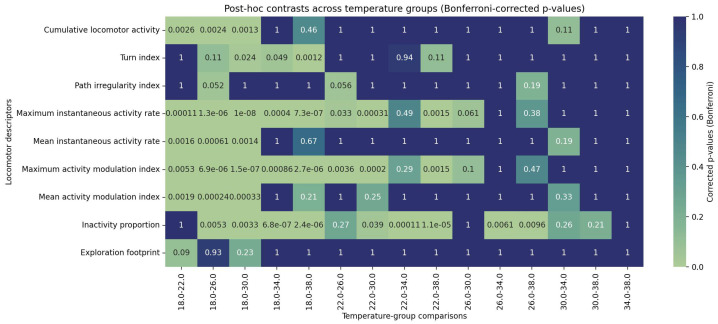
Bonferroni-corrected post hoc *p*-values for pairwise comparisons among temperature groups across the locomotor descriptors extracted from the motion-tracking system. Lower *p*-values indicate the descriptors and temperature contrasts that contributed most strongly to the statistical separation of the thermal conditions, with the clearest effects concentrated in maximum instantaneous activity rate, maximum activity modulation index, and inactivity proportion.

The post hoc comparisons accompanied this interpretation. Maximum instantaneous activity rate, maximum activity modulation index, and inactivity proportion showed the clearest separation among temperature groups in both the T-value contrast heatmap (Figure 12) and the Bonferroni-corrected significance heatmap (Figure 13). The strongest contrasts were concentrated around the transition toward 30 °C. Inactivity proportion was especially notable because it retained significant or near-significant contrasts across several temperature comparisons, consistent with a possible stable increase in immobility at higher temperatures. In contrast, cumulative locomotor activity, exploration footprint, and mean dynamic descriptors indicated a more heterogeneous pairwise patterns.

The post hoc results suggest that the main thermal effect was a restructuring of locomotor organization across the temperature gradient. Intermediate conditions, particularly around 30 °C, were associated with a favorable combination of high activity and strong modulation, whereas higher temperatures shifted behavior toward preserved peak output but reduced continuity and greater inactivity. Thus, temperature appeared to influence not only how much the animals moved, but also how locomotor effort was distributed over time.

### 3.4. Spearman Correlations

The interval-based Spearman analysis further supported a non-linear thermal organization in *G. portentosa* locomotion (Table 3). In the lower-to-intermediate interval (18–30 °C), all locomotor descriptors showed significant positive correlations with temperature. The strongest associations were observed for maximum instantaneous activity rate (ρ=0.445, p<0.0001), maximum activity modulation index (ρ=0.432, p<0.0001), and inactivity proportion (ρ=0.342, p<0.0001), indicating that warming within this range strengthened peak locomotor output and temporal modulation while also increasing immobility.

Above 30 °C, the correlation structure largely disappeared. Between 30 and 38 °C, inactivity proportion was the only descriptor that remained significantly correlated with temperature, and the association was weak (ρ=0.178, p=0.0469; Table 3). Several descriptors shifted toward weak negative coefficients, including cumulative locomotor activity, path irregularity index, mean instantaneous activity rate, mean activity modulation index, and exploration footprint. Across the full 18–38 °C range, significant correlations remained mainly for inactivity proportion (ρ=0.449, p<0.0001), maximum instantaneous activity rate (ρ=0.385, p<0.0001), maximum activity modulation index (ρ=0.373, p<0.0001), and turn index (ρ=0.202, p=0.0013). These results are consistent with the mixed-effects and post hoc analyses and support the interpretation of a functional turning region around 30 °C.

### 3.5. Effects of Body Length and Sex

**Figure 14:**
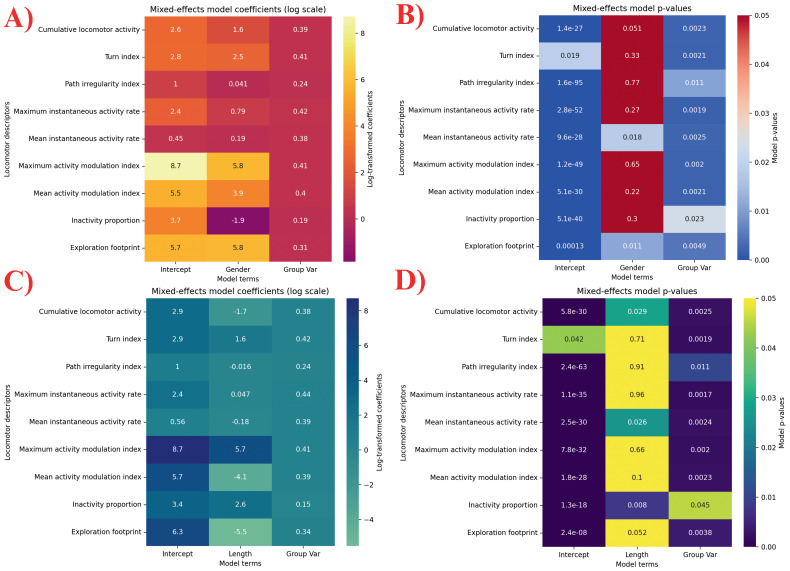
Mixed-effects model results for population-related variables. (**A**) Log-scaled model coefficients for sex. (**B**) Corresponding model *p*-values for sex. (**C**) Log-scaled model coefficients for body length. (**D**) Corresponding model *p*-values for body length. Body length showed moderate effects for a limited subset of locomotor descriptors, whereas sex showed weaker and less consistent effects across the dataset.

The auxiliary mixed-effects analysis of body length and sex suggested that neither variable accounted for the main thermal pattern observed in locomotor performance. Body length was related to moderate effects in a limited subset of descriptors, particularly those associated with integrated locomotor output and sustained movement, whereas sex seemed to exert only a weak and inconsistent influence across the dataset (Figure 14). In the original binary coding of the models, body length was divided at a threshold of 6 cm, and this factor supported its clearest associations with cumulative locomotor activity, mean instantaneous activity rate, and inactivity proportion. The overall direction of the body-length effect suggested that shorter individuals tended to exhibit reduced locomotor output, lower average movement rates, and a greater tendency toward interrupted or less sustained locomotion. Although these effects were detectable at the model level, they were clearly smaller and less coherent than those associated with temperature, indicating that body size might have contributed to inter-individual variability without defining the principal structure of the thermal response.

By contrast, sex showed limited explanatory value for most locomotor descriptors. The results indicate that sex did not reach robust statistical significance across the majority of the models, although weak positive tendencies were observed in variables related to exploration and locomotor intensity, including exploration footprint, mean instantaneous activity rate, and some acceleration-related descriptors in the original nomenclature. In practical terms, this suggests that males and females may differ subtly in certain aspects of movement organization, but these differences were not strong enough to support a clear sex-based interpretation of the behavioral patterns observed here. Because neither sex nor body length reached sufficient statistical strength, no post hoc or correlation analyses were pursued for these variables, and their effects were treated as secondary sources of variability rather than as principal explanatory factors.

These results indicate that the characteristic locomotor profile observed across the temperature gradient may not be attributed to population structure alone. Instead, body length and sex appear to contribute only modestly to the dispersion of the data, while temperature seems to remain the dominant factor shaping locomotor behavior. Although morphological and demographic variability may influence the magnitude of movement in individual animals, they do not seem to explain the suggested non-linear thermal pattern centered around 30 °C. Thus, the effects of body length and sex might be regarded as secondary modifiers of locomotor output, relevant for future refinements in experimental design but seem to be insufficient to account for the main temperature-dependent response documented here.

Across the thermal gradient, locomotion in *Gromphadorhina portentosa* supported a non-linear organization, providing evidence of a favorable overall profile concentrated around 30 °C and a possible deterioration of sustained performance at higher temperatures. This pattern was most consistently expressed in parameters related to peak locomotor output and behavioral arrest, whereas broader integrated parameters such as cumulative locomotor activity or exploration footprint captured the net outcome of competing tendencies with lower statistical sharpness.

Results for population-related variables must be further examined as previous studies in cockroaches have reported sexual dimorphism in body traits, with females often being larger or heavier than males, and have also suggested that larger males may display competitive advantages associated with body size [42,43]. Nevertheless, in the present dataset, neither body length nor sex seemed to exhibit sufficient explanatory strength to account for the non-linear thermal profile suggested here.

## 4. Discussion

Statistical analysis supports the hypothesis that *G. portentosa* locomotion might be significantly influenced by temperature but in a non-linear manner as might be expected. Specifically, locomotion seems to be statistically affected only within the low to mid-temperature range (18 °C–30 °C). Beyond 30 °C, locomotion not only seems to fail to improve but also begins to decline, as observed in most of the locomotion variables for the 34 °C group.

This non-linear pattern is consistent with the general expectation for ectothermic animals, in which locomotor and physiological performance often increases with temperature within a favorable range but declines or becomes less stable once thermal conditions approach stressful levels [2,3,6,7,8]. Therefore, the apparent transition around 30 °C may represent not only a point of high locomotor output but also suggests a possible functional boundary beyond which sustained locomotor organization becomes less stable.

It remains uncertain whether this characteristic behavior is attributable to stress induced by exposure to high temperatures or to other factors, as our results for sex and body length did not show sufficient statistical significance to explain this specific behavior. Although body length is not an accurate proxy for cockroach age, it may serve as an estimator, which is particularly relevant for studying thermal effects in this species, since thermal tolerance in *G. portentosa* has been shown to be greater in adult cockroaches than in younger individuals [44].

Further research is required to determine which physiological and behavioural mechanisms underlie the non-linear thermal modulation of *G. portentosa* locomotion observed in the present study. Our results suggest that locomotor performance improves from low to intermediate temperatures, reaching a possible favorable overall profile around 30 °C, but that this improvement is not maintained at higher temperatures. Instead, warmer conditions, particularly near 34 °C, were associated with increased inactivity and a more fragmented locomotor organization. This pattern indicates that high temperature did not simply suppress locomotion, but altered how locomotor effort was distributed over time. This interpretation agrees with previous studies in other insects, including *Acheta domesticus*, *Drosophila*, and *Sitona gressorius*, where temperature has been shown to affect locomotor activity, speed, and overall performance [30,31,32]. However, the present results suggest that in *G. portentosa*, the most informative effect of temperature may not be only the total amount of movement, but the way locomotor activity is temporally organized into sustained activity, bursts, and inactivity.

This interpretation is consistent with the idea that thermal effects in *G. portentosa* may be process-specific rather than uniformly expressed across all physiological systems. For example, Harrison et al. reported that temperature did not significantly alter the respiratory response of *G. portentosa* under different thermal conditions [45]. Similarly, McCue and De Los Santos examined whether oxygen delivery becomes limiting as body temperature increases and found that changes in oxygen availability did not consistently improve survival except when oxygen was completely absent [44]. These findings suggest that the locomotor changes detected here are unlikely to be explained only by respiratory limitation or oxygen availability.

Instead, the observed decline here in sustained locomotor organization above the intermediate thermal range may reflect other mechanisms, including neuromuscular performance, behavioral stress responses, fatigue, dehydration risk, or cellular-level stress. Such mechanisms are plausible because temperature can influence multiple components of insect performance simultaneously, including metabolic rate, neuromuscular function, sensory processing, and behavioral responsiveness [2,4,6]. Thus, the increased inactivity observed at warmer conditions could emerge from the combined effect of several physiological and behavioral processes rather than from a single limiting factor. Evidence from another cockroach species, *Diploptera punctata*, supports this broader view, as thermal conditions were shown to modify DNA methylation patterns, with lower methylation variation at 28 °C, interpreted as a possible indication of an optimal developmental temperature [46]. Although this evidence comes from a different species and addresses molecular rather than locomotor responses, it suggests that cockroaches may respond to thermal variation through mechanisms that are not directly respiratory.

Therefore, the present results should not be interpreted as evidence that temperature has a simple positive or negative effect on *G. portentosa* locomotion. Rather, temperature appears to reorganize locomotor output as warming up to approximately 30 °C seems to enhance activity intensity and locomotor responsiveness, similar to the 28 °C suggested optimal developmental temperature for *Diploptera punctata* [46], whereas further warming seems to preserve short bursts of activity while reducing sustained locomotion and increasing inactivity. Future studies should therefore integrate locomotor, physiological, and molecular measurements, as well as acclimation history, humidity, hydration state, and neuromuscular variables, to provide a more complete explanation of locomotor and other physiological processes in *G. portentosa*.

Additionally, subsequent investigations should take into account the temperature effects reported here, as our results suggest that velocity, acceleration, and, to some extent, total travel might be significantly influenced by thermal variations. For instance, further studies may benefit from the reduced locomotor activity observed at 34 °C if the objective is to test whether some sort of stimulation enhances *G. portentosa* locomotion. Conversely, other studies may find it advantageous to use moderate temperatures, such as 30 °C, if the aim is to optimize cockroach locomotion as much as possible. This consideration is particularly important for cyborg, biohybrid, and stimulation-based studies using *G. portentosa* because environmentally driven changes in baseline locomotion could modify the apparent effectiveness of neural, sensory, or onboard stimulation protocols [18,19,20,21,33]. Therefore, temperature should be treated not only as a background experimental condition, but as an active modulator of locomotor responsiveness.

It is also important that future work considers other environmental variables affecting *G. portentosa* locomotion, as factors like noise and human presence were not controlled, and while light intensity was stabilized, its potential effects remain insufficiently understood.

The complementary illumination assays also indicate a possible additional biologically relevant indication that *Gromphadorhina portentosa* locomotion might be modulated by controlled visual conditions. The most interesting aspect is the qualitative difference among light conditions: UV illumination was associated with producing the clearest positive modulation of locomotor output, whereas green light seemed to induce smaller shifts and the no-light control remained closer to baseline. This pattern suggests that not all visual stimuli engage locomotor behavior with the same strength and is consistent with the broader idea that spectral composition can shape insect behavioral responses rather than light acting only as a generic on/off stimulus [24,47]. This interpretation is supported by studies showing that light effects on insects are strongly wavelength- and species-dependent. For example, green light negatively affects locomotion and growth in *Nilaparvata lugens* [9], light exposure modifies locomotor and reproductive traits in *Orius sauteri* [10], UV illumination inhibits locomotion in *Periplaneta americana* [24], and blue and red light produce opposite behavioral effects in *Blatta lateralis* [26]. In addition, visual responses in cockroaches can depend on stimulus intensity and spatial properties [27]. Therefore, the stronger UV-associated modulation observed here should be interpreted as possible preliminary evidence of wavelength-sensitive locomotor modulation in *G. portentosa*.

In the case of *G. portentosa*, these results should still be interpreted cautiously because the illumination experiment was designed as a proof-of-concept sensitivity assay rather than as a population-level characterization due to the small sample size (N = 12).

Also, illumination assays were not phase-order counterbalanced: UV and green trials began with a no-light baseline followed by the assigned second phase. Therefore, time-dependent effects such as habituation, fatigue, or spontaneous changes in activity cannot be completely separated from illumination-associated effects. For this reason, the illumination results were interpreted only as exploratory within-subject responses.

The shift in locomotion observed in our preliminary results under UV light suggests that short-wavelength light may represent a particularly salient environmental cue for eliciting behavioral change, a possibility that deserves direct testing in future work under larger sample sizes and more specialized visual protocols. For instance, a previous study showed that environmental factors such as light play an important role in the welfare of other similar cockroach species as *Gromphadorhina oblongonota*, inducing stress and anxiety when they are unable to escape from light [48].

This possibility is relevant because light stimulation has been proposed as a non-invasive strategy for modulating cockroach locomotion, including in approaches using UV light [28]. However, future experiments should also evaluate whether repeated exposure reduces responsiveness, since habituation to light exposure has been reported in other cockroach species such as *Eublaberus posticus* [29].

Future illumination experiments should separate the effects of wavelength, intensity, exposure duration, and repeated stimulation. This is important because cockroach visual responses can depend on stimulus brightness and spatial properties [27] and because repeated light exposure may produce habituation in some species [29]. Therefore, although the present exploratory assay suggests that UV illumination may modulate *G. portentosa* locomotion, a full characterization will require larger sample sizes, balanced wavelength conditions, controlled irradiance levels, and repeated-exposure protocols.

Methodologically, these results also emphasize the importance of quantifying locomotion under stable and reproducible environmental conditions. Previous studies have noted that locomotor variability, inconsistent responsiveness, and differences among measurement systems can complicate the interpretation of insect locomotor responses [22,23,33,34]. In this context, the present platform was useful not as the main focus of the study, but as a controlled framework that allowed temperature- and illumination-associated locomotor changes to be detected under synchronized environmental monitoring. However, it still has the limitation of only acquiring data of tethered treadmill locomotion instead of free-walking behavior which is more desirable.

Further studies should compare these results with open-arena locomotion data acquired under controlled environmental conditions in order to capture additional aspects of freely expressed walking behavior. Future work should also evaluate the usability of this system with other insect species, considering that the current implementation is limited to individuals with sufficient strength to move the polystyrene ball and with an adequate flat area on the pronotum for Velcro attachment. Nevertheless, miniaturized versions of this system could potentially be adapted for smaller insect species, such as *Blattella germanica* or members of the family *Gryllidae*.

## 5. Conclusions

In this study, the effects of programmed chamber temperature on treadmill-based locomotor output were evaluated in 42 Madagascar hissing cockroaches (*G. portentosa*). The results suggest a non-linear thermal dependence, with a possible favorable overall locomotor profile around the programmed 30 °C chamber condition and reduced sustained performance at higher temperatures. The descriptors most consistently associated with temperature were those related to peak locomotor output and inactivity, indicating that warming may initially enhance locomotor capacity but may later promote a more fragmented and less sustained behavioral pattern.

Complementary illumination assays were performed as exploratory proof-of-concept experiments. Under the present treadmill conditions, UV illumination was associated with the clearest preliminary shift in locomotor output, but this result should be interpreted cautiously because of the small sample size and the non-counterbalanced phase order.

Overall, the proposed platform provides an accessible framework for recording treadmill-based locomotor output under controlled temperature and illumination conditions. Future work should further validate the system against open-arena behavior, larger sample sizes, controlled irradiance measurements, and additional physiological variables.

## Figures and Tables

**Table 1:** Internal illumination LED specifications according to the manufacturer (STEREN).

Light	Current	Voltage	λ	Angle
Red	20 mA	1.8 V	620 nm	18°
Green	20 mA	2.8 V	515 nm	18°
Blue	20 mA	2.8 V	465 nm	18°
UV	16 mA	3.4 V	390–400 nm	30°

**Table 2:** Descriptive statistics of the locomotor descriptors across temperature groups. Each entry is reported as mean ± standard deviation for the corresponding temperature condition.

Parameter	Temperature Condition					
	18 °C (n = 42)	22 °C (n = 42)	26 °C (n = 42)	30 °C (n = 41)	34 °C (n = 42)	38 °C (n = 42)
Cumul. act. [cm]	10.51 ± 5.96	14.87 ± 8.47	15.34 ± 8.66	17.62 ± 11.27	12.43 ± 9.62	15.07 ± 11.69
Mean act. rate [cm s^−1^]	0.47 ± 0.27	0.67 ± 0.40	0.69 ± 0.39	0.79 ± 0.51	0.57 ± 0.42	0.65 ± 0.49
Act. mod. rate [cm s^−2^]	186.59 ± 98.37	268.83 ± 155.19	283.83 ± 147.91	332.45 ± 203.76	245.35 ± 177.22	278.35 ± 215.06
Inact. prop. [%]	19.57 ± 21.19	24.51 ± 25.24	34.54 ± 21.69	39.97 ± 25.19	53.02 ± 29.89	52.31 ± 27.16
Exp. footprint [cm^2^]	262.94 ± 455.30	459.72 ± 696.98	396.47 ± 428.88	518.84 ± 938.34	281.54 ± 406.89	488.91 ± 817.12
Max. act. rate [cm s^−1^]	6.18 ± 2.77	8.67 ± 3.62	10.24 ± 4.37	11.72 ± 4.97	10.80 ± 5.66	12.05 ± 5.17
Max. act. mod. [cm s^−2^]	3978.38 ± 1925.40	5275.79 ± 2374.09	6462.38 ± 2768.72	7463.62 ± 3507.55	6867.18 ± 3695.54	7638.04 ± 3518.82
Path irreg. [a.u.]	1.55 ± 0.55	1.61 ± 0.53	1.89 ± 0.63	1.77 ± 0.70	1.87 ± 1.16	1.63 ± 0.60
Turn index [a.u.]	0.02 ± 40.45	8.20 ± 56.08	20.36 ± 45.32	25.12 ± 53.06	25.21 ± 46.84	32.54 ± 54.01

**Table 3:** Spearman rank correlation coefficients (ρ) and corresponding *p*-values for locomotor descriptors across the lower-to-intermediate thermal interval (18–30 °C), the upper thermal interval (30–38 °C), and the complete thermal gradient (18–38 °C). The table highlights a strong and coherent positive correlation structure below 30 °C, in contrast to the marked reduction of thermal association above that range, where inactivity proportion remained the only significant correlate.

Locomotor Descriptor	18–30 °C ρ	18–30 °C *p*-Value	30–38 °C ρ	30–38 °C *p*-Value	18–38 °C ρ	18–38 °C *p*-Value
Cumulative locomotor activity	0.249	0.0012	−0.116	0.1992	0.051	0.4170
Turn index	0.188	0.0150	0.065	0.4708	0.202	0.0013
Path irregularity index	0.163	0.0353	−0.071	0.4287	0.050	0.4307
Maximum instantaneous activity rate	0.445	<0.0001	0.039	0.6688	0.385	<0.0001
Mean instantaneous activity rate	0.266	0.0005	−0.139	0.1219	0.056	0.3809
Maximum activity modulation index	0.432	<0.0001	0.023	0.7955	0.373	<0.0001
Mean activity modulation index	0.297	0.0001	−0.130	0.1488	0.080	0.2072
Inactivity proportion	0.342	<0.0001	0.178	0.0469	0.449	<0.0001
Exploration footprint	0.198	0.0103	−0.114	0.2058	−0.004	0.9444

## Data Availability

A video showing the overall development of the methodological platform as well as some of the individuals using it can be seen via YouTube through this link: https://www.youtube.com/watch?v=9OEWWu4eFy4, accessed on 9 June 2026. The complete dataset for the thermal-dependent locomotion experiments is freely accessible on Zenodo through the following link: https://zenodo.org/records/16882893, accessed on 9 June 2026. Part of the dataset for light-dependent locomotion experiments can also be accessed by Zenodo through this link: https://zenodo.org/records/17958716, accessed on 9 June 2026. The GUI files for motion-tracking and environmental control are accessible through this link: https://zenodo.org/records/17941650, accessed on 9 June 2026. The STL files for the spherical treadmill can be accessed through this link: https://zenodo.org/records/17918217, accessed on 9 June 2026. Additionally, a simplified CLI tool for environmental control and motion-tracking can be accessed through: https://zenodo.org/records/16888053, accessed on 9 June 2026.

## Supplementary Materials

The thermal characterization of the system can be seen through this public document: https://doi.org/10.5281/zenodo.20573702.
